# Three twisted tales: A case series of caecal volvulus

**DOI:** 10.1016/j.ijscr.2024.109776

**Published:** 2024-05-19

**Authors:** Bisola Salaja, Mobarak Kunna, Aisling Hogan

**Affiliations:** a Department of Colorectal Surgery, University Hospital Galway, Newcastle Rd, Galway H91 YR71, Ireland; b School of Medicine, College of Medicine, Nursing and Health Sciences, University of Galway, Ireland

**Keywords:** Case series, Caecal volvulus, Laparoscopic right hemicolectomy, Acute abdomen

## Abstract

**Introduction:**

Caecal volvulus is a form of intestinal obstruction with life-threatening potential. While rare, it represents a perilous aetiology of intestinal blockage, with clinical manifestations spanning from abdominal discomfort to mortality.

**Case series:**

We report the cases of three young adults (two males and one female) who presented to the emergency department with different manifestations of severe abdominal pain. All occurred within one month in a tertiary referral centre. Radiological evaluations confirmed the diagnosis of caecal volvulus in all. Subsequently, these individuals underwent right hemicolectomies with end-to-end anastomosis. All experienced an uncomplicated perioperative course.

**Clinical discussion:**

Caecal volvulus is uncommon, but its yearly incidence is increasing. Early detection and a heightened level of suspicion lead to a timely diagnosis, reducing morbidity and mortality rates.

**Conclusion:**

We report a case series of caecal volvulus, emphasizing its variable presentation and highlighting the critical importance of an early diagnosis. Typically, patients have a history of similar episodes that resolve without any medical intervention. Definitive treatment involves right hemicolectomy while conservative management is associated with very high recurrence rates. Early detection allows for prompt intervention, resulting in reduced morbidity and mortality rates.

## Highlights

• Patients with caecal volvulus typically have a history of similar episodes that resolved spontaneously.
• Outdated statistical data can lead physicians not to consider a particular diagnosis in their clinical assessments.
• Early detection allows for prompt intervention, resulting in reduced morbidity and mortality rates.

## 1 Introduction

Caecal volvulus (CV) is a rare form of intestinal obstruction with life-threatening potential. It involves the rotation or torsion of the mobile caecum and ascending colon.

**Fig. 1:**
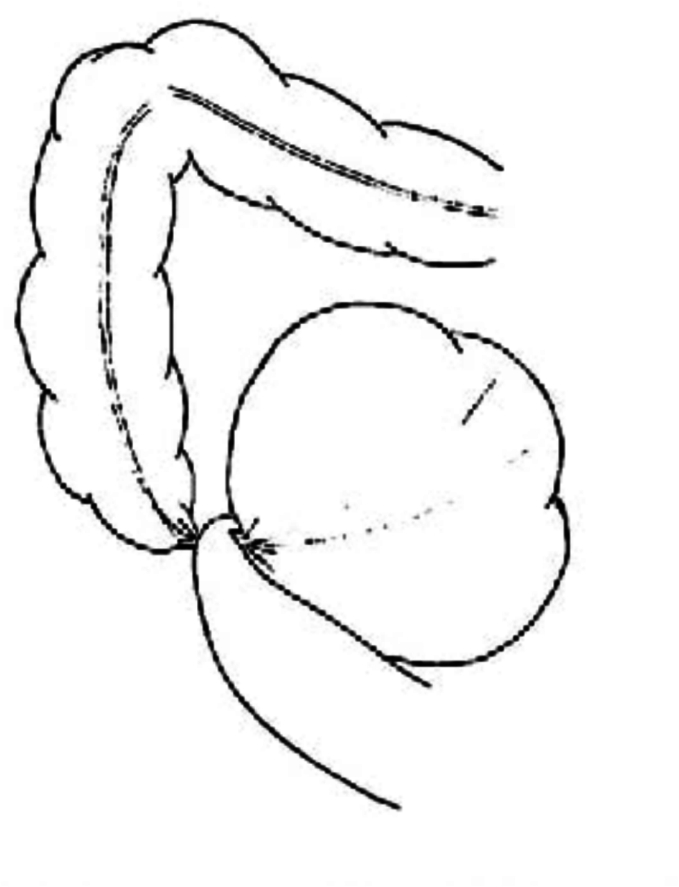
Type 1 Caecal Volvulus [2]

**Fig. 2:**
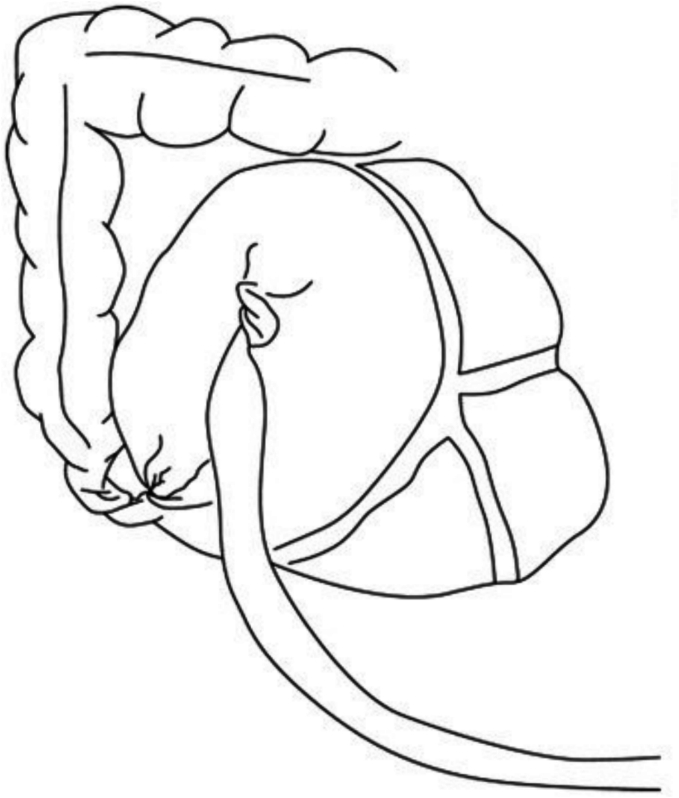
Type 2 Caecal Volvulus [3]

**Fig. 3:**
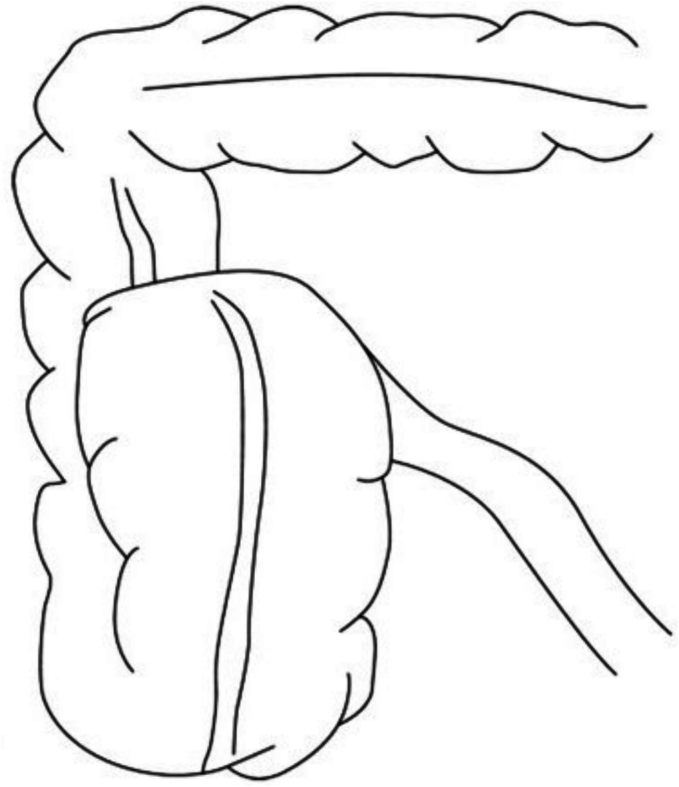
Type 3 Caecal Volvulus [3].

There are three known classifications of CV [1] (Fig. 1, Fig. 2, Fig. 3):

• **Type 1:** Caecum undergoes clockwise axial torsion along its long axis, and the volvulized cecum remains in the right lower quadrant.

• **Type 2:** Caecum and terminal ileum are implicated and positioned ectopically in the left upper quadrant (LUQ).

• **Type 3:** Caecal bascule entails the upward folding of the caecum rather than axial twisting.

In 80 % of CV cases, patients commonly present with Type 1 and Type 2, with the remaining 20 % experiencing Type 3 folding referred to as Caecal Bascule [1,4]. The primary aetiology is of embryological origin, involving abnormal mesentery fixation leading to excessive caecal mobility [1].

CV has traditionally been deemed rare, with an incidence rate of 2.8–7.1 cases per year per 1 million individuals often cited [5]. It's important to highlight that this data stems from a 1949 paper, a period predating the computerized tomography (CT) scan era, which is now the gold standard for diagnosis [6].

Despite its historical paucity, we present three documented cases of caecal volvulus within the service of a single colorectal surgeon, in an academic tertiary hospital, occurring in close proximity, a mere month apart. Our objective is to raise awareness of the importance of considering this diagnosis more frequently when confronted with an unclear acute abdomen. These three cases are presented sequentially.

The management of these patients was in accordance with the Clinical Practice Guidelines of the American Society of Colon and Rectal Surgeons. This case series has been reported in line with the PROCESS guidelines [7].

## 2 Case series

### 2.1 Case 1

A 51-year-old male self-presented to the emergency department (ED) with a sudden onset of severe abdominal pain localized in the epigastric region. Prior medical history was unremarkable. He was previously asymptomatic and described the pain as constant, sharp, and of unbearable intensity (8–9/10), with no signs of radiation. The pain was accompanied by recurrent vomiting, anorexia, and chills. He had experienced similar episodes in the past which spontaneously resolved. The absence of lower urinary tract symptoms and the lack of a family history of inflammatory bowel disease were observed. The patient had a medical history of hypercholesterolemia and was being treated with a stable dose of Atorvastatin (10 mg).

Physical examination indicated the patient's distressed condition, with significant tenderness in the epigastric region and high-pitched bowel sounds. Laboratory tests revealed a slightly elevated white blood cell count (9.2 × 10^9^ /L), normal haemoglobin levels (14.3 g/dL), a normal C-reactive protein levels (0.9 mg/L), and a normal venous lactate level (0.9 mmol/L).

**Fig. 4:**
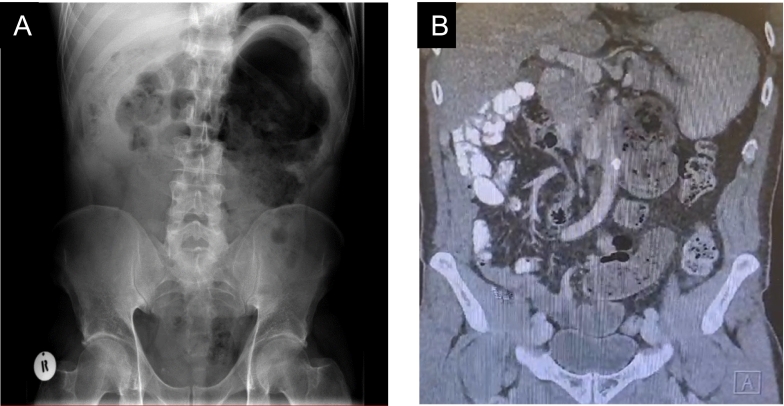
Abdominal Xray and CT abdomen pelvis of Case 1.

Imaging studies, including an abdominal X-ray and subsequent CT abdomen pelvis, demonstrated dilated bowel loops in the LUQ and the absence of bowel in the lower quadrants (Fig. 4A) indicating an unusual location of the patient's cecum in the LUQ, as well as a whirl sign in the CT (Fig. 4B), consistent with a diagnosis of caecum ectopia and suggestive of CV.

**Fig. 5:**
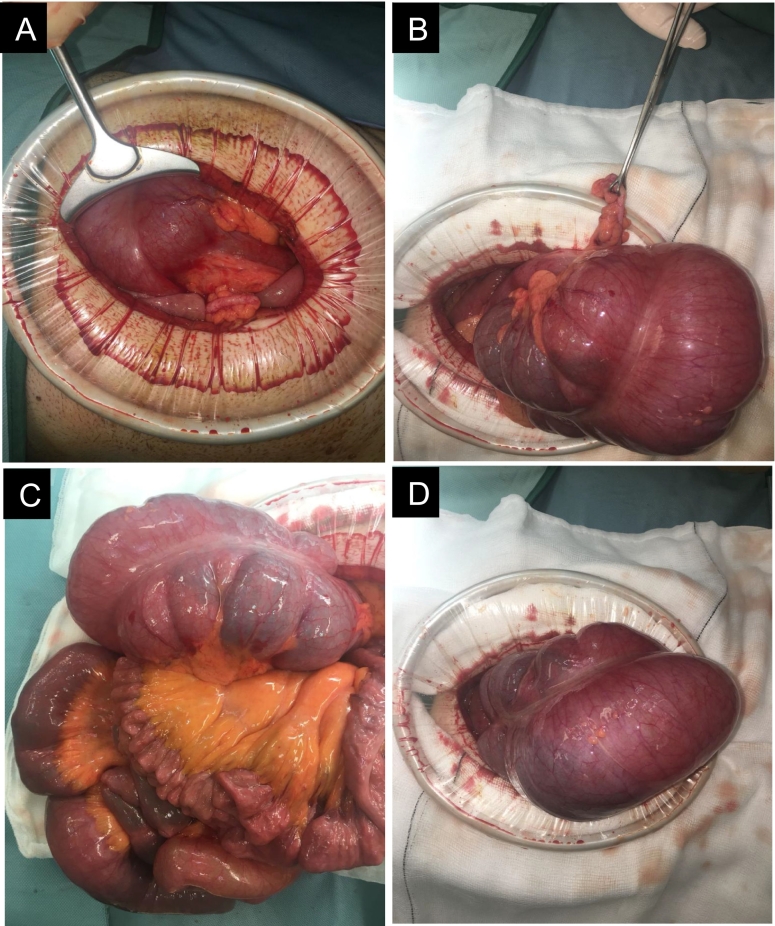
Intraoperative findings of Case 1.

The patient underwent immediate preoperative resuscitation and preparation, followed by an emergency laparotomy and subsequent right hemicolectomy. Intraoperative findings confirmed the distended (Fig. 5C&D) and dislocated cecum in the LUQ (Fig. 5A), with the appendix visible for size comparison and orientation (Fig. 5B). Intraoperatively, the bowel was meticulously inspected, revealing an absence of any discernible evidence indicative of perforation. Furthermore, no manifestations suggestive of intestinal malrotation in other regions were observed. An effective end-to-end anastomosis was performed post-resection. He experienced an uneventful postoperative course, with a six-day inpatient stay.

There were no complications or adverse outcomes. He continued to be in good health during the subsequent outpatient assessment post-operatively.

### 2.2 Case 2

A 48-year-old female self-presented to the ED with a one-day history of sudden onset severe abdominal pain localized in the right iliac fossa. Her prior medical history was unremarkable. She reported waking up with a sharp, constant pain across the lower abdomen associated with nausea, and resistant to analgesia. Similar episodes in the past led to investigations for irritable bowel syndrome (IBS). However, she denied altered bowel habits, lower urinary tract symptoms, or red flag symptoms. She was a non-smoker, consumed minimal alcohol, and had a history of two vaginal deliveries.

On examination, her abdomen was tender and mildly distended in the RIF. She exhibited associated rebound tenderness in the right iliac fossa and no other significant findings. She was in obvious physical distress. Laboratory investigations revealed a normal white blood cell count, haemoglobin level and C-reactive protein level. A raised venous lactate level (2.6 mmol/L), and a negative serum ßhcG was also noted.

**Fig. 6:**
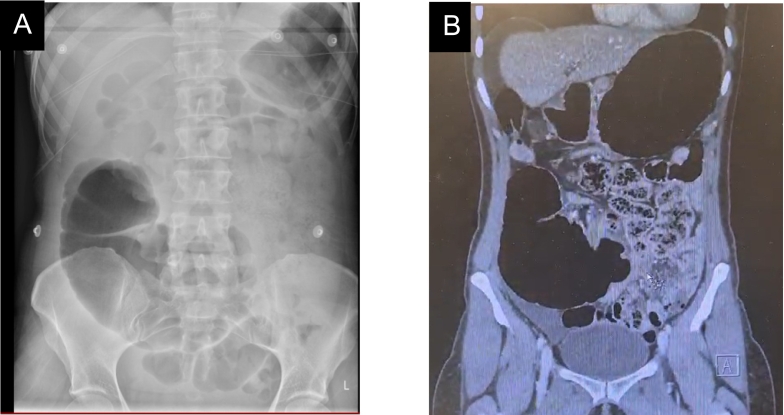
Abdominal Xray and CT abdomen pelvis of Case 2.

Abdominal x-ray demonstrating marked distension of the caecum (Fig. 6A) and CT abdomen pelvis (Fig. 6B) showing similar findings.

**Fig. 7:**
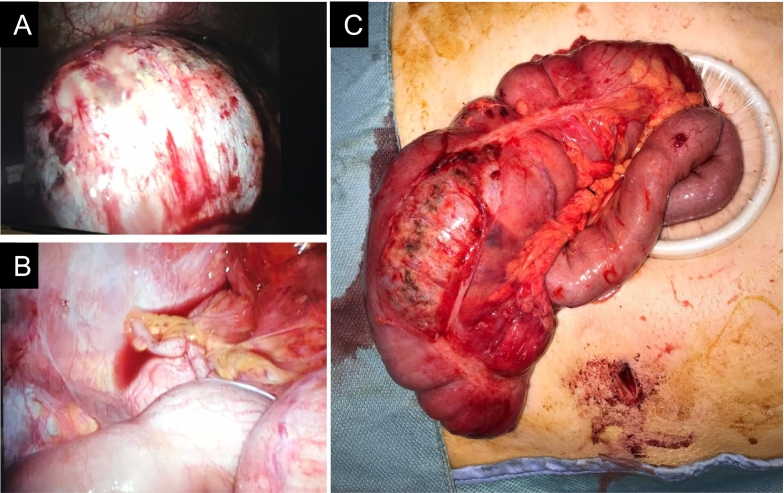
Intraoperative findings of Case 2.

She subsequently underwent immediate preoperative resuscitation and preparation, followed by an emergency laparoscopic right hemicolectomy. Through laparoscopic visualization, the markedly distended caecum was observed (Fig. 7A), accompanied by free fluid in the pelvic region and an uninflamed appendix (Fig. 7B). Intraoperative findings confirmed a grossly distended, discoloured, oedematous caecum with impending perforation and serosal involvement (Fig. 7C). While a minor volume of pelvic fluid was present, no indications of perforation were detected. An ileocolic anastomosis was therefore formed. She too had an uneventful postoperative course, with a five-day inpatient stay and the same follow-up.

Timely laparoscopic intervention proved effective, offering advantages in terms of reduced postoperative recovery time and cosmetic outcomes (Fig. 7). She continued to be in good health during the subsequent outpatient assessment six-weeks post-operatively.

### 2.3 Case 3

The final case in this series detailed a similar perioperative course to the previous cases, wherein a 35-year-old male with an otherwise unremarkable medical history presented to the ED with severe sudden-onset abdominal pain. Notably, he had experienced a nearly identical episode one year prior, which had resolved spontaneously. The current episode did not respond to analgesia, and the patient reported one bowel movement on the morning of the presentation, with no altered bowel habits or lower urinary tract symptoms (LUTS) previously.

**Fig. 8:**
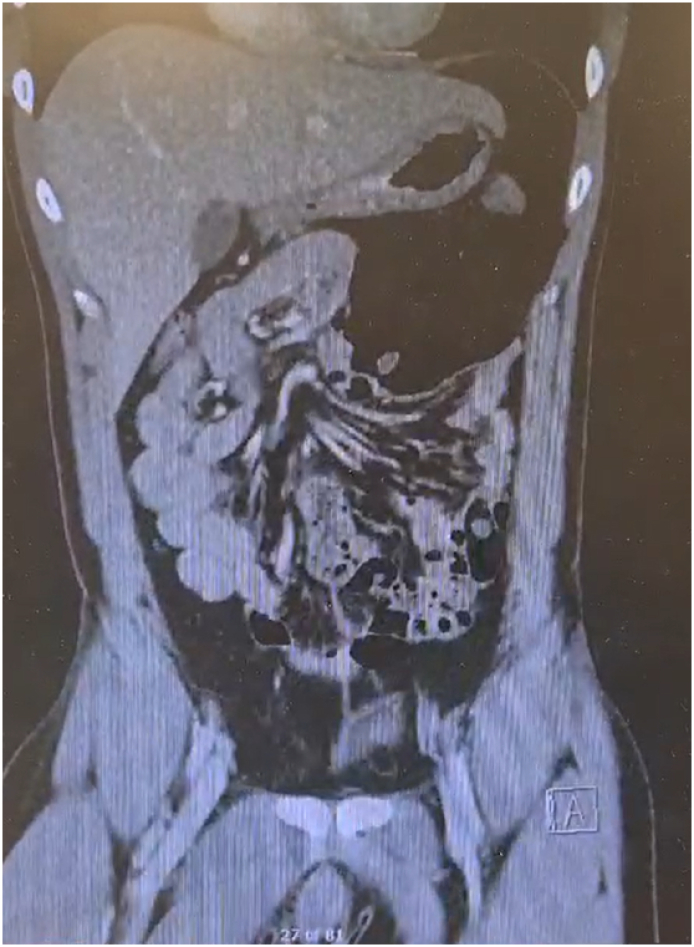
CT abdomen pelvis of Case 3.

Laboratory investigations demonstrated unremarkable blood results. CT abdomen pelvis revealed distended loops of bowel, with the caecum translocated in the LUQ. Notably, a whirl sign was observed in this instance (Fig. 8)., akin to the previous case. A diagnosis of Type 2 caecal volvulus was confirmed. Intraoperatively there were no signs of perforation and no intestinal malrotation observed in other areas. Subsequently, the patient underwent a laparoscopic right hemicolectomy, experiencing an uncomplicated postoperative course with a five-day inpatient stay.

## 3 Discussion

The presented case series highlights the variable and unsuspecting presentations of CV, emphasizing the importance of maintaining a high level of suspicion for this condition, particularly in cases with recurrent abdominal pain. Notably, patients in this series often had prior episodes that resolved spontaneously, underscoring the elusive nature of CV and the challenge it poses in early diagnosis.

CV, though traditionally considered a relatively rare condition [8], demands vigilant consideration when called to an acute abdomen. The findings in this case series indicate a potentially higher incidence than previously acknowledged, as events unfolded in close succession. A large multistate retrospective study carried out in the United States of America supports an increase in the incidence of CV [9].

Several factors may contribute to the escalating incidence of caecal volvulus. These include an aging population, increased prevalence of chronic constipation [10], changes in dietary habits leading to altered bowel motility [11], and improved diagnostic modalities resulting in better detection rates. Furthermore, the rise in comorbidities such as obesity and connective tissue disorders may predispose individuals to this condition [12].

Prevention of caecal volvulus primarily involves addressing these predisposing factors and promoting bowel health. Strategies aimed at preventing chronic constipation through dietary modifications [13], adequate hydration [13], and regular exercise may reduce the risk of volvulus occurrence, given the high prevalence of constipation and bowel dysfunctions among affected individuals [14].

Patients experiencing repeated, albeit transient, episodes of severe abdominal pain should prompt a thorough investigation, as demonstrated in the cases discussed. The primary imaging modality for diagnosing CV is CT abdomen and pelvis [6], with the pathognomonic “whirl sign” [15,16] manifesting as observable twists in the mesenteric vessels.

Definitive management for CV, as evidenced by the cases in this series, involves a right hemicolectomy [8,17]. While alternative options have been considered in the past, such as reduction through methods like barium enema or colonoscopy [6], the consistently high recurrence rates [18] associated with these procedures highlight the advantage of right hemicolectomy in preventing relapses. Additionally, these non-resectional approaches pose the potential for hazardous delays, including bowel perforation, before surgery, consequently elevating the mortality risk [1]. Given advancements in surgical techniques and its additional benefits, laparoscopic right hemicolectomy has become the preferred primary treatment option [6,19].

## 4 Conclusion

In conclusion, this case series reinforces the need for a nuanced clinical approach and a high index of suspicion in diagnosing CV, particularly when faced with unsuspecting presentations and a history of recurrent, spontaneously resolving abdominal pain. The recurrent nature of episodes serves as a valuable clinical marker, and the definitive management through right hemicolectomy underscores the importance of surgical intervention in achieving successful outcomes and preventing recurrence. Healthcare providers should remain vigilant in not ruling out CV in cases presenting similarly to other common acute abdominal emergencies. This series contributes to the existing knowledge base, shedding light on the diverse clinical spectrum and optimal management strategies for this infrequent yet potentially severe condition. Further research into the underlying aetiology and efficacy of preventive strategies is warranted to mitigate the burden of this potentially life-threatening condition.

## Consent for publication

All patients detailed in this case series provided informed consent.

## Ethical approval

This case series publication did not strictly meet the criteria of research. Although illustrative, it does not meet the Federal Policy for the Protection of Human Subjects definition of Research, which requires an investigation that contributes to generalizable knowledge about a disease or condition. Subsequently my supervisor discussed with ethics board and they deemed not necessary for a formal submission.

## Funding

NIL.

## Author contribution

Dr Bisola Salaja - Data curation, Writing – original draft, Writing – review & editing

Mr Mobarak Kunna – Data curation

Prof Aisling Hogan – Conceptualisation, Supervision, Writing – review & editing

## Guarantor

Prof Aisling Hogan

## Research registration number

1.Name of the registry:

NIL.

2.Unique identifying number or registration ID:

NIL.

3.Hyperlink to your specific registration (must be publicly accessible and will be checked):

NIL.

## Conflict of interest statement

NIL.
